# *N*,*N*′-Bis(3-bromo-2-hy­droxy­benzyl­idene)propane-1,3-di­amine

**DOI:** 10.1107/S1600536813025403

**Published:** 2013-09-25

**Authors:** Xiao-Zhen Wang

**Affiliations:** a College of Chemistry, Dalian University of Technology, Dalian 116024, People’s Republic of China

## Abstract

In the title compound, C_17_H_16_Br_2_N_2_O_2_, the dihedral angle between the benzene rings is 57.7 (3)°. The conformation of the central N—C—C—C—N chain is *gauche*-*anti* [torsion angles = −64.2 (4) and −167.8 (4)°]. Two intra­molecular O—H⋯N hydrogen bonds occur. In the crystal, molecules are linked by pairs of C—H⋯O hydrogen bonds, forming inversion dimers.

## Related literature

For a related structure, see: Elerman *et al.* (1998 ▶).
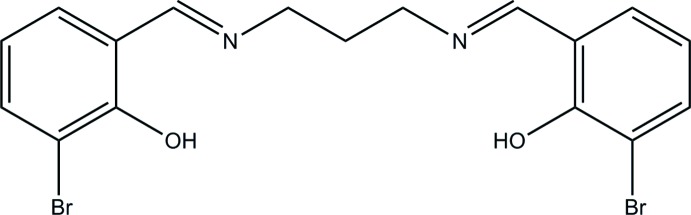


## Experimental

### 

#### Crystal data

C_17_H_16_Br_2_N_2_O_2_
*M* *_r_* = 440.14
Monoclinic, 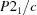

*a* = 12.779 (1) Å
*b* = 10.1894 (8) Å
*c* = 14.3953 (12) Å
β = 113.744 (2)°
*V* = 1715.8 (2) Å^3^
*Z* = 4
Mo *K*α radiation
μ = 4.74 mm^−1^
*T* = 298 K
0.23 × 0.22 × 0.22 mm

#### Data collection

Bruker SMART CCD diffractometer
Absorption correction: multi-scan (*SADABS*; Bruker, 2007 ▶) *T*_min_ = 0.409, *T*_max_ = 0.422
19018 measured reflections
3728 independent reflections
2669 reflections with *I* > 2σ(*I*)
*R*_int_ = 0.055

#### Refinement

*R*[*F* ^2^ > 2σ(*F* ^2^)] = 0.054
*wR*(*F* ^2^) = 0.141
*S* = 1.07
3728 reflections
210 parameters
H-atom parameters constrained
Δρ_max_ = 0.73 e Å^−3^
Δρ_min_ = −1.01 e Å^−3^

### 

Data collection: *SMART* (Bruker, 2007 ▶); cell refinement: *SAINT* (Bruker, 2007 ▶); data reduction: *SAINT*; program(s) used to solve structure: *SHELXTL* (Sheldrick, 2008 ▶); program(s) used to refine structure: *SHELXTL*; molecular graphics: *SHELXTL*; software used to prepare material for publication: *SHELXTL*.

## Supplementary Material

Crystal structure: contains datablock(s) global, I. DOI: 10.1107/S1600536813025403/hb7137sup1.cif

Structure factors: contains datablock(s) I. DOI: 10.1107/S1600536813025403/hb7137Isup2.hkl

Supplementary material file. DOI: 10.1107/S1600536813025403/hb7137Isup3.cml

Additional supplementary materials:  crystallographic information; 3D view; checkCIF report

## Figures and Tables

**Table 1:** Hydrogen-bond geometry (Å, °)

*D*—H⋯*A*	*D*—H	H⋯*A*	*D*⋯*A*	*D*—H⋯*A*
O1—H1⋯N1	0.82	1.83	2.567 (4)	148
O2—H2⋯N2	0.82	1.82	2.553 (5)	149
C7—H7⋯O2^i^	0.93	2.60	3.490 (5)	161

Symmetry code: (i) 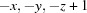.

**Fig. 1.**
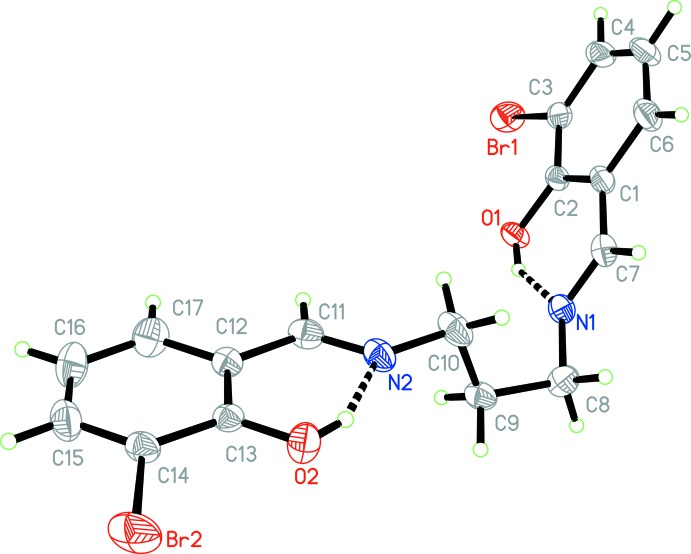
The molecular structure of the title compound with ellipsoids drawn at the 30% probability level.

## supplementary crystallographic information

### 1. Experimental

3-Bromosalicylaldehyde (1 mmol, 0.20 g) and propane-1,3-diamine (0.5 mmol, 0.037 g) were dissolved and stirred in 50 ml methanol at room temperature. The resulting yellow solution was kept in air for a few days, generating yellow blocks as the solvent slowly evaporated.

### 2. Refinement

H atoms were placed in idealized positions and constrained to ride on their parent atoms, with C—H distances of 0.93-0.97 Å, O—H distances of 0.82 Å, and with *U*_iso_(H) = 1.2*U*_eq_(C) and 1.5*U*_eq_(O).

### Crystal data

**Table:**

C_17_H_16_Br_2_N_2_O_2_	*F*(000) = 872
*M**_r_* = 440.14	*D*_x_ = 1.704 Mg m^−^^3^
Monoclinic, *P*2_1_/*c*	Mo *K*α radiation, λ = 0.71073 Å
*a* = 12.779 (1) Å	Cell parameters from 8352 reflections
*b* = 10.1894 (8) Å	θ = 2.5–27.9°
*c* = 14.3953 (12) Å	µ = 4.74 mm^−^^1^
β = 113.744 (2)°	*T* = 298 K
*V* = 1715.8 (2) Å^3^	Block, yellow
*Z* = 4	0.23 × 0.22 × 0.22 mm

### Data collection

**Table:**

Bruker SMART CCD diffractometer	3728 independent reflections
Radiation source: fine-focus sealed tube	2669 reflections with *I* > 2σ(*I*)
Graphite monochromator	*R*_int_ = 0.055
ω scans	θ_max_ = 27.0°, θ_min_ = 2.5°
Absorption correction: multi-scan (*SADABS*; Bruker, 2007)	*h* = −16→16
*T*_min_ = 0.409, *T*_max_ = 0.422	*k* = −13→12
19018 measured reflections	*l* = −18→18

### Refinement

**Table:**

Refinement on *F*^2^	Primary atom site location: structure-invariant direct methods
Least-squares matrix: full	Secondary atom site location: difference Fourier map
*R*[*F*^2^ > 2σ(*F*^2^)] = 0.054	Hydrogen site location: inferred from neighbouring sites
*wR*(*F*^2^) = 0.141	H-atom parameters constrained
*S* = 1.07	*w* = 1/[σ^2^(*F*_o_^2^) + (0.0633*P*)^2^ + 2.4024*P*] where *P* = (*F*_o_^2^ + 2*F*_c_^2^)/3
3728 reflections	(Δ/σ)_max_ = 0.001
210 parameters	Δρ_max_ = 0.73 e Å^−^^3^
0 restraints	Δρ_min_ = −1.01 e Å^−^^3^

### Special details

**Table:**

Geometry. All esds (except the esd in the dihedral angle between two l.s. planes) are estimated using the full covariance matrix. The cell esds are taken into account individually in the estimation of esds in distances, angles and torsion angles; correlations between esds in cell parameters are only used when they are defined by crystal symmetry. An approximate (isotropic) treatment of cell esds is used for estimating esds involving l.s. planes.
Refinement. Refinement of F^2^ against ALL reflections. The weighted R-factor wR and goodness of fit S are based on F^2^, conventional R-factors R are based on F, with F set to zero for negative F^2^. The threshold expression of F^2^ > 2sigma(F^2^) is used only for calculating R-factors(gt) etc. and is not relevant to the choice of reflections for refinement. R-factors based on F^2^ are statistically about twice as large as those based on F, and R- factors based on ALL data will be even larger.

### Fractional atomic coordinates and isotropic or equivalent isotropic displacement parameters (Å^2^)

**Table:**

	*x*	*y*	*z*	*U*_iso_*/*U*_eq_	
Br1	0.30295 (5)	0.46157 (5)	0.10435 (4)	0.0628 (2)	
Br2	−0.37545 (5)	0.26857 (7)	0.63883 (5)	0.0760 (2)	
N1	0.0868 (3)	0.1060 (3)	0.2505 (2)	0.0387 (8)	
N2	−0.0781 (3)	0.2245 (4)	0.4529 (3)	0.0454 (9)	
O1	0.1442 (2)	0.2886 (3)	0.1573 (2)	0.0433 (7)	
H1	0.1017	0.2399	0.1714	0.065*	
O2	−0.2045 (3)	0.1922 (3)	0.5504 (3)	0.0514 (8)	
H2	−0.1583	0.1739	0.5261	0.077*	
C1	0.2813 (3)	0.1666 (4)	0.2944 (3)	0.0394 (9)	
C2	0.2528 (3)	0.2639 (4)	0.2194 (3)	0.0365 (9)	
C3	0.3399 (4)	0.3351 (4)	0.2097 (3)	0.0421 (10)	
C4	0.4525 (4)	0.3144 (5)	0.2741 (4)	0.0562 (13)	
H4	0.5100	0.3641	0.2671	0.067*	
C5	0.4797 (4)	0.2202 (6)	0.3486 (4)	0.0644 (15)	
H5	0.5556	0.2072	0.3927	0.077*	
C6	0.3956 (4)	0.1454 (5)	0.3583 (4)	0.0560 (12)	
H6	0.4148	0.0803	0.4076	0.067*	
C7	0.1924 (4)	0.0872 (4)	0.3054 (3)	0.0417 (10)	
H7	0.2132	0.0209	0.3539	0.050*	
C8	0.0013 (4)	0.0262 (4)	0.2677 (4)	0.0472 (11)	
H8A	0.0393	−0.0419	0.3168	0.057*	
H8B	−0.0477	−0.0159	0.2048	0.057*	
C9	−0.0711 (4)	0.1100 (5)	0.3066 (3)	0.0440 (10)	
H9A	−0.1066	0.1798	0.2585	0.053*	
H9B	−0.1315	0.0563	0.3112	0.053*	
C10	−0.0019 (4)	0.1693 (6)	0.4094 (3)	0.0546 (12)	
H10A	0.0462	0.1024	0.4544	0.065*	
H10B	0.0472	0.2378	0.4026	0.065*	
C11	−0.1054 (4)	0.3460 (5)	0.4441 (3)	0.0440 (10)	
H11	−0.0739	0.4017	0.4111	0.053*	
C12	−0.1838 (3)	0.3993 (4)	0.4838 (3)	0.0372 (9)	
C13	−0.2308 (3)	0.3159 (4)	0.5356 (3)	0.0353 (9)	
C14	−0.3080 (4)	0.3746 (5)	0.5709 (3)	0.0429 (10)	
C15	−0.3355 (4)	0.5048 (5)	0.5559 (4)	0.0594 (13)	
H15	−0.3870	0.5400	0.5799	0.071*	
C16	−0.2879 (5)	0.5853 (5)	0.5054 (4)	0.0679 (15)	
H16	−0.3074	0.6737	0.4953	0.081*	
C17	−0.2116 (5)	0.5328 (5)	0.4708 (4)	0.0580 (13)	
H17	−0.1779	0.5865	0.4383	0.070*	

### Atomic displacement parameters (Å^2^)

**Table:**

	*U*^11^	*U*^22^	*U*^33^	*U*^12^	*U*^13^	*U*^23^
Br1	0.0638 (4)	0.0634 (4)	0.0714 (4)	−0.0147 (2)	0.0379 (3)	0.0024 (2)
Br2	0.0673 (4)	0.1079 (5)	0.0731 (4)	−0.0240 (3)	0.0495 (3)	−0.0080 (3)
N1	0.0385 (19)	0.043 (2)	0.0394 (18)	0.0032 (16)	0.0210 (16)	−0.0008 (15)
N2	0.0368 (19)	0.061 (3)	0.0401 (19)	0.0046 (17)	0.0171 (16)	−0.0039 (17)
O1	0.0286 (15)	0.0499 (18)	0.0470 (16)	0.0027 (13)	0.0107 (13)	0.0083 (13)
O2	0.068 (2)	0.0393 (17)	0.064 (2)	0.0023 (15)	0.0438 (17)	0.0047 (14)
C1	0.032 (2)	0.047 (2)	0.035 (2)	0.0082 (18)	0.0099 (18)	−0.0050 (18)
C2	0.031 (2)	0.042 (2)	0.037 (2)	0.0031 (17)	0.0148 (18)	−0.0086 (17)
C3	0.040 (2)	0.044 (2)	0.048 (2)	−0.0009 (19)	0.023 (2)	−0.0100 (19)
C4	0.036 (2)	0.067 (3)	0.068 (3)	−0.004 (2)	0.024 (2)	−0.025 (3)
C5	0.028 (2)	0.079 (4)	0.072 (4)	0.009 (2)	0.006 (2)	−0.021 (3)
C6	0.042 (3)	0.063 (3)	0.052 (3)	0.017 (2)	0.006 (2)	−0.005 (2)
C7	0.047 (3)	0.043 (2)	0.037 (2)	0.011 (2)	0.0186 (19)	0.0043 (18)
C8	0.056 (3)	0.045 (3)	0.049 (3)	−0.006 (2)	0.030 (2)	−0.005 (2)
C9	0.036 (2)	0.054 (3)	0.045 (2)	−0.004 (2)	0.0192 (19)	−0.0015 (19)
C10	0.038 (2)	0.085 (4)	0.043 (2)	0.010 (2)	0.018 (2)	−0.008 (2)
C11	0.041 (2)	0.057 (3)	0.038 (2)	−0.006 (2)	0.0207 (19)	0.0004 (19)
C12	0.036 (2)	0.043 (2)	0.0305 (19)	0.0002 (18)	0.0116 (17)	0.0011 (17)
C13	0.033 (2)	0.041 (2)	0.0299 (19)	−0.0037 (17)	0.0106 (17)	−0.0052 (16)
C14	0.037 (2)	0.056 (3)	0.038 (2)	−0.006 (2)	0.0189 (19)	−0.0071 (19)
C15	0.052 (3)	0.069 (3)	0.054 (3)	0.015 (3)	0.020 (2)	−0.013 (2)
C16	0.081 (4)	0.045 (3)	0.078 (4)	0.019 (3)	0.032 (3)	0.004 (3)
C17	0.071 (3)	0.046 (3)	0.058 (3)	0.005 (2)	0.027 (3)	0.013 (2)

### Geometric parameters (Å, º)

**Table:**

Br1—C3	1.899 (5)	C7—H7	0.9300
Br2—C14	1.883 (4)	C8—C9	1.521 (6)
N1—C7	1.274 (5)	C8—H8A	0.9700
N1—C8	1.462 (5)	C8—H8B	0.9700
N2—C11	1.279 (6)	C9—C10	1.511 (6)
N2—C10	1.465 (5)	C9—H9A	0.9700
O1—C2	1.337 (5)	C9—H9B	0.9700
O1—H1	0.8200	C10—H10A	0.9700
O2—C13	1.300 (5)	C10—H10B	0.9700
O2—H2	0.8200	C11—C12	1.443 (6)
C1—C6	1.394 (6)	C11—H11	0.9300
C1—C2	1.402 (6)	C12—C17	1.400 (6)
C1—C7	1.454 (6)	C12—C13	1.415 (6)
C2—C3	1.382 (6)	C13—C14	1.412 (6)
C3—C4	1.378 (6)	C14—C15	1.366 (7)
C4—C5	1.376 (8)	C15—C16	1.388 (8)
C4—H4	0.9300	C15—H15	0.9300
C5—C6	1.369 (8)	C16—C17	1.370 (8)
C5—H5	0.9300	C16—H16	0.9300
C6—H6	0.9300	C17—H17	0.9300
			
C7—N1—C8	119.2 (4)	C10—C9—H9A	109.1
C11—N2—C10	122.1 (4)	C8—C9—H9A	109.1
C2—O1—H1	109.5	C10—C9—H9B	109.1
C13—O2—H2	109.5	C8—C9—H9B	109.1
C6—C1—C2	119.7 (4)	H9A—C9—H9B	107.8
C6—C1—C7	119.9 (4)	N2—C10—C9	110.2 (4)
C2—C1—C7	120.3 (4)	N2—C10—H10A	109.6
O1—C2—C3	119.7 (4)	C9—C10—H10A	109.6
O1—C2—C1	121.7 (4)	N2—C10—H10B	109.6
C3—C2—C1	118.6 (4)	C9—C10—H10B	109.6
C4—C3—C2	121.2 (4)	H10A—C10—H10B	108.1
C4—C3—Br1	119.8 (4)	N2—C11—C12	122.1 (4)
C2—C3—Br1	119.0 (3)	N2—C11—H11	119.0
C5—C4—C3	119.9 (5)	C12—C11—H11	119.0
C5—C4—H4	120.1	C17—C12—C13	121.0 (4)
C3—C4—H4	120.1	C17—C12—C11	119.5 (4)
C6—C5—C4	120.4 (4)	C13—C12—C11	119.5 (4)
C6—C5—H5	119.8	O2—C13—C14	121.6 (4)
C4—C5—H5	119.8	O2—C13—C12	122.2 (4)
C5—C6—C1	120.2 (5)	C14—C13—C12	116.2 (4)
C5—C6—H6	119.9	C15—C14—C13	121.8 (4)
C1—C6—H6	119.9	C15—C14—Br2	119.8 (4)
N1—C7—C1	121.8 (4)	C13—C14—Br2	118.4 (3)
N1—C7—H7	119.1	C14—C15—C16	121.1 (5)
C1—C7—H7	119.1	C14—C15—H15	119.4
N1—C8—C9	110.9 (3)	C16—C15—H15	119.4
N1—C8—H8A	109.5	C17—C16—C15	119.1 (5)
C9—C8—H8A	109.5	C17—C16—H16	120.5
N1—C8—H8B	109.5	C15—C16—H16	120.5
C9—C8—H8B	109.5	C16—C17—C12	120.7 (5)
H8A—C8—H8B	108.0	C16—C17—H17	119.7
C10—C9—C8	112.5 (4)	C12—C17—H17	119.7

### Hydrogen-bond geometry (Å, º)

**Table:**

*D*—H···*A*	*D*—H	H···*A*	*D*···*A*	*D*—H···*A*
O1—H1···N1	0.82	1.83	2.567 (4)	148
O2—H2···N2	0.82	1.82	2.553 (5)	149
C7—H7···O2^i^	0.93	2.60	3.490 (5)	161

Symmetry code: (i) −*x*, −*y*, −*z*+1.
